# Shunning the scoop: Sidestepping the race to publish

**DOI:** 10.1016/j.isci.2022.104080

**Published:** 2022-04-04

**Authors:** Christina Lynggaard, Joanne E. Littlefair, Kristine Bohmann, Elizabeth L. Clare

**Affiliations:** 1 Section for Evolutionary Genomics, Globe Institute, Faculty of Health and Medical Sciences, University of Copenhagen, 1353 Copenhagen, Denmark; 2 Department of Biology, York University, 4700 Keele Street, Toronto, ON M3J 1P3 Canada; 3 School of Biological and Behavioural Sciences, Queen Mary University of London, E1 4NS London, UK

## Abstract

What happens when a researcher finds out that research very similar to their own is already being conducted? What if they find out that the said research is also very close to being published? First, there is probably anxiety and panic. Maybe, there are frantic calls to collaborators. Perhaps Twitter rants about the phenomenon of scooping that plagues all researchers, especially those early-career researchers who often feel they are in a race to get their best work out to the world.

**Figure:**
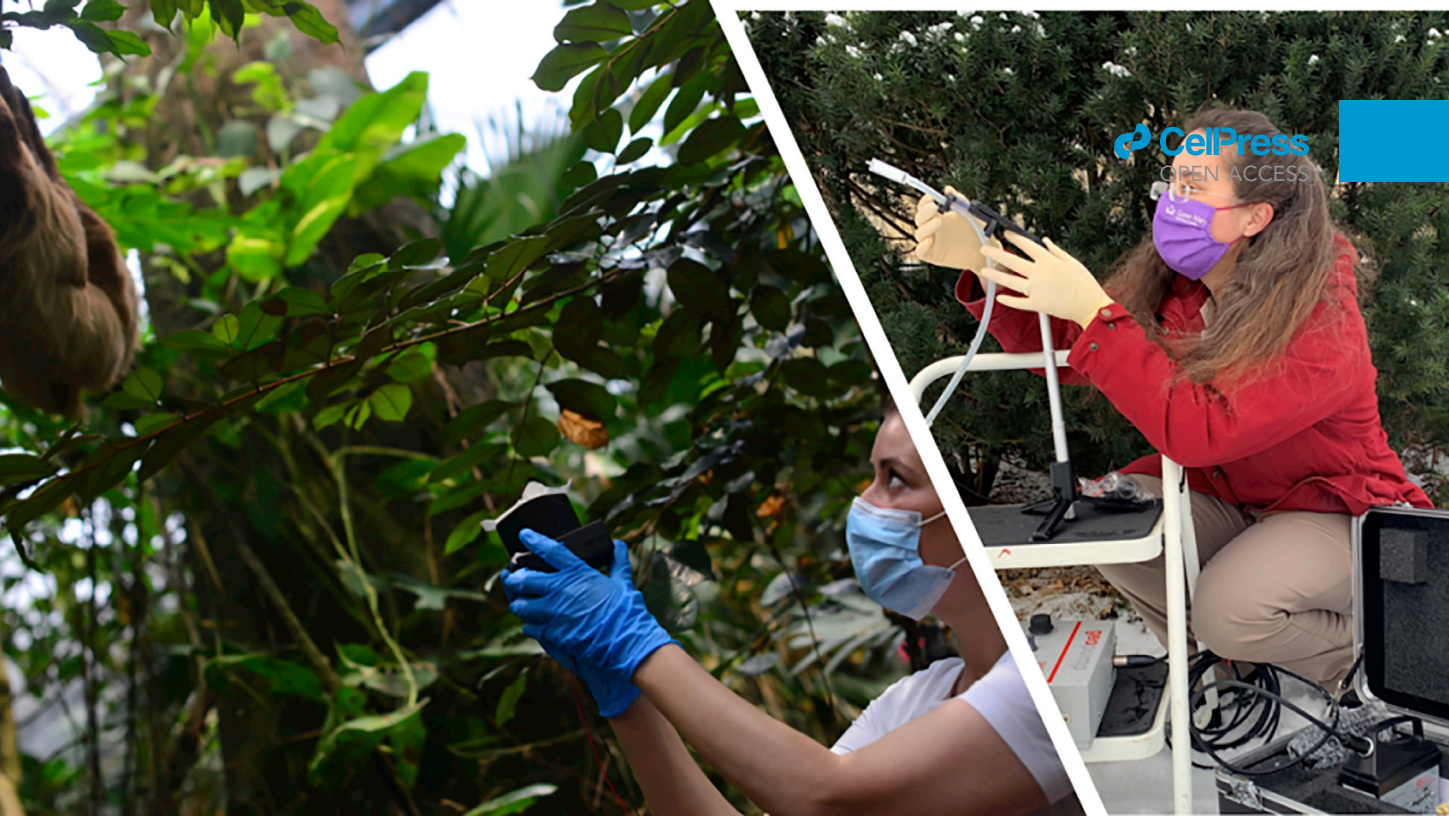
Above image: (Left) Kristine Bohmann demonstrates collection of air samples nearby a sloth at Copenhagen Zoo; (Right) Elizabeth Clare samples air to collect airborne DNA

I’ve never seen such a convergence of research. In a fast moving research field you have to expect you will eventually scoop someone on an idea and be scooped in return. But none of us had ever seen such a perfectly timed case. It was true independent scientific replication in every sense of the phrase.

I think the ‘gentle(wo)mans’ agreement we made between the two teams when we became aware of each other’s studies meant a lot to both teams as we ventured into this very unfamiliar territory of coordinating article submission, peer-review, publication, and press with a team that we at first had seen as competitors.

Scooping someone on an idea is not fun. In a race, someone has to lose. We did not want anyone involved to be in that situation. Working together was better for the research and better for all the people involved.

I remember people not talking about their results at conferences or keeping parts of their methods hidden so they wouldn’t be scooped. I didn’t ever receive any input about how positive working with your “rivals” could be.

In this Backstory, we discuss with the authors of two recent *Current Biology* works (Clare et al., 2022; Lynggaard et al., 2022) about what happened when they became aware of their concurrent studies happening at the same time in two different countries related to airborne DNA and the prospects of using it to monitor biodiversity.

Instead of racing to publish their works to “beat” out the other group, they simply reached out to each other and made the agreement that they would coordinate the publication process, so that each of their independent works could get the publicity it deserved. The authors discuss here how they shunned the scoop and worked together to bring forward two independent, but complementary, pieces pushing forward the field of airborne DNA, as well as offering advice to other researchers who might find themselves in a similar precarious situation.

### Proximity

#### How did you embark upon this project? Who were the players in each research team?

Elizabeth L. Clare (York University/Queen Mary University of London): It was a coincidence of events that led to the project for the UK team. I was commissioned by the UK Environment Agency to write a “think piece” on the use of DNA to monitor biodiversity in the terrestrial biome (Environmental Agency, 2021). In this document I described all the sources of eDNA on land and listed “air” because I just assumed it was used. I went looking for case studies for my report and was very surprised how little was out there. I found a couple of reports about plant material in dust and some work on pathogens but for animal life on land it seemed to be all speculation. It was a couple of months after this, that our institution in London advertised a fund for “high risk, high impact” ideas. My group had a history of working on novel ways to collect eDNA, so I contacted some likely partners and end users from industry, government regulators, and academic colleagues, and we made a pitch to the grant funding committee.

**Figure:**
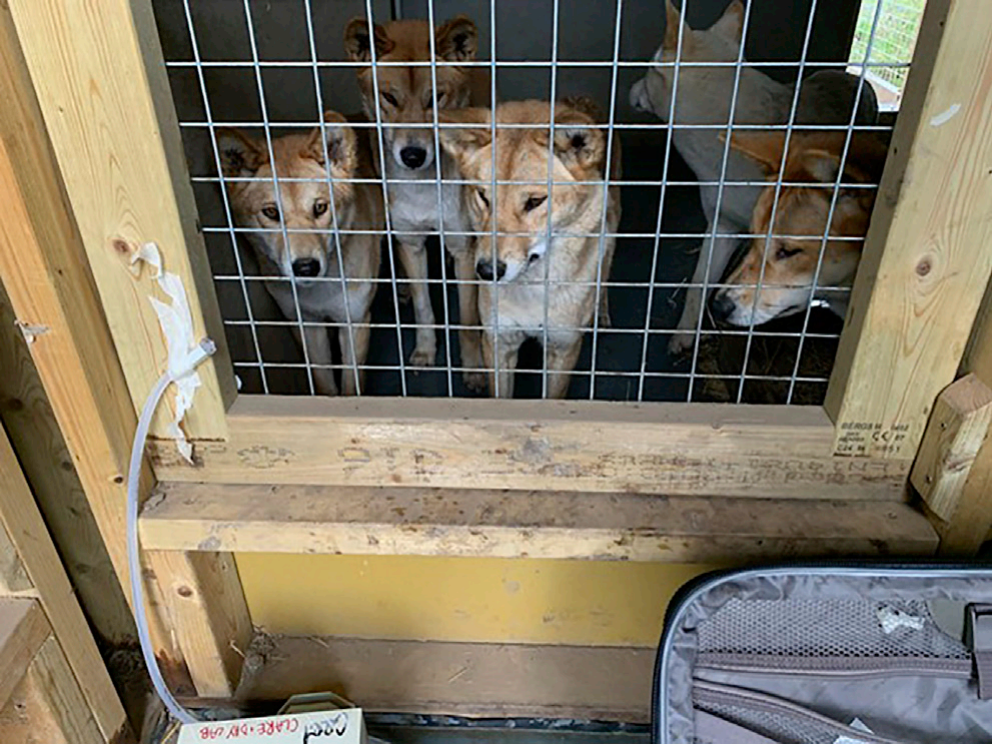
Dingos at the zoo eye air sampling equipment with curiosity. Credit: Elizabeth Clare

Joanne E. Littlefair (Queen Mary University of London): Our earlier proof of concept study was a risky project. We didn’t necessarily think that we could sequence DNA out of thin air, but we thought that a laboratory population of mammals would give us the best chance of doing so. After that success (Clare et al., 2021), we knew we wanted to scale up to a seminatural system using non-native animals, so a zoo was a perfect next step (Clare et al., 2022). Beth is really good at pulling together a team with different strengths, so for the zoo project we had technicians, a postdoc, members of staff from the zoo and me doing the bioinformatic and statistical analyses. I was on maternity leave while we were doing this project, so the analyses were conducted while my daughter had naps or went down to sleep in the evenings.

Kristine Bohmann (University of Copenhagen): Our work was also supported by a high risk-high gain grant, the Danish VILLUM Foundation. They have a funding instrument called VILLUM Experiment, which, in their own words: “support the ideas which have a limited chance of succeeding, but hold great potential if they do”. In 2018, we applied for this grant for a project where we suggested using airborne environmental DNA to monitor terrestrial vertebrates. But following peer-review, our proposal was rejected. We reapplied the following year, were awarded the grant, and started the project in early 2020.

We quickly realized that although we have knowledge of environmental DNA in my group at Globe Institute at University of Copenhagen, we had limited knowledge about bioaerosols and vacuuming animal DNA out of thin air. We therefore started collaborating with Professor Matthew Johnson and his student who works on air particles. Further, we established a collaboration with Professor and Zoological Director of Copenhagen Zoo, Mads Frost Bertelsen, who contributed with essential knowledge about the zoo, the animals kept there, and animal conservation efforts.

Christina Lynggaard (University of Copenhagen): This project was very interdisciplinary as we needed people with knowledge about environmental DNA, air particles, zoo animals, and conservation efforts. We were very fortunate to end up with a very good group of researchers who were interested in the study in Copenhagen. Although we started collecting samples in Copenhagen Zoo in September 2020, we were in the middle of the COVID pandemic and got the results in Spring (2021).

#### How did you become aware of each other’s work? How did you bring everyone together?

Clare: The coordination bewteen the two teams happened much later in the project. We had completed our work, written our paper, and posted it on a preprint server when we became aware of the “other paper”. We were shocked to discover that Kristine and Christina had done the same experiment at the same time and written almost the same paper. It was astonishing. You could have swapped paragraphs between them and not noticed it. I’ve never seen such a convergence of research. In a fast moving research field you have to expect you will eventually scoop someone on an idea and be scooped in return. But none of us had ever seen such a perfectly timed case. It was true independent scientific replication in every sense of the phrase.

**Figure:**
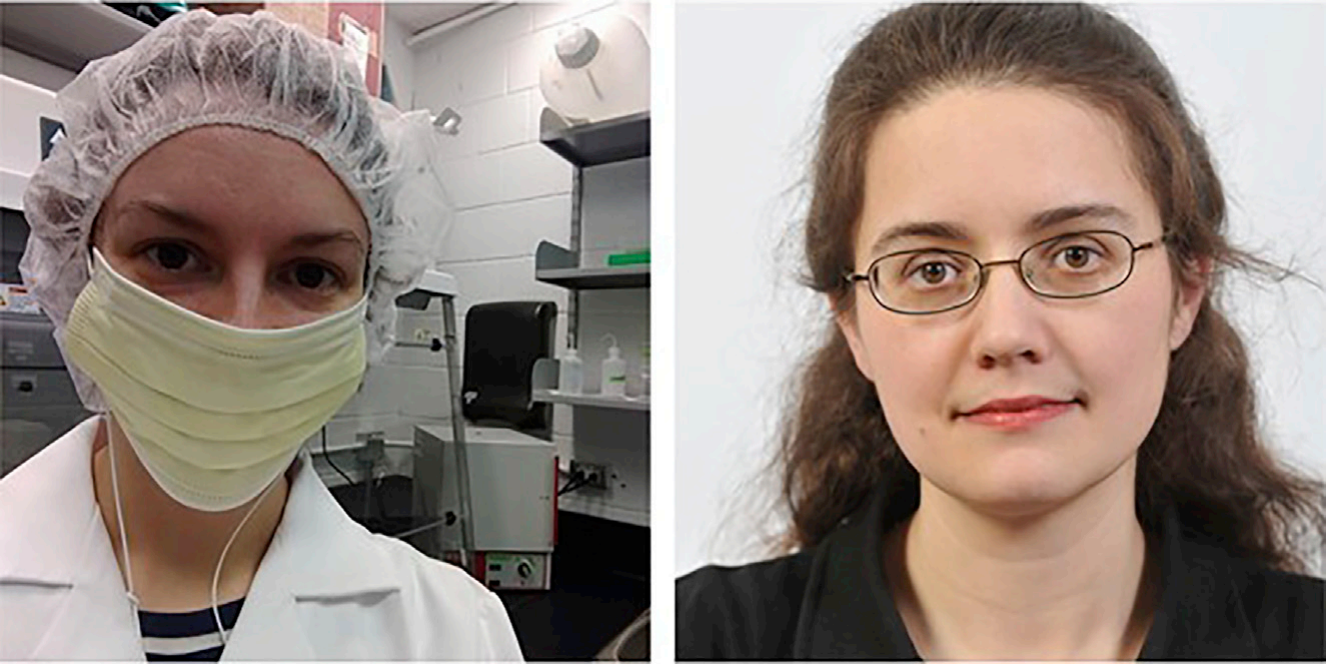
(Left) Joanne Littlefair in eDNA lab gear and (right) headshot of Elizabeth Clare

We were left with a choice: have a race to see who could publish first or find a different way forward. We had the benefit of knowing each other. Kristine and I had published together before. We called them up on Zoom and as a group we decided that we did not want to compete for publication and we were actually happy to see perfect replication of the “crazy idea.” It made it easier to feel confident about what we were doing, when we realized someone else had found exactly the same thing. We saw this as an asset rather than a competition. We needed to be unorthodox about our attempt to publish, but the potential for success in the long run could be high.

Bohmann: We got the first results in the Spring of 2021, and we could not believe our own eyes! We thought that our first sampling in Copenhagen Zoo would be for us to test out three air filtering samplers, sampling times, air volumes, etc. Besides, we thought that we would use this to get a hint on which direction we should go for optimizing the sampling. We never thought we would get such robust results in the first go! After an intense period of analyses and writing it all up, we were ready to submit the article.

Literally two days before we planned to submit, we were contacted by colleagues saying that there was another study out in preprint very similar to ours. We were shocked! I reached out to several colleagues who I knew had experienced competition regarding publication of research results in the past to get their perspective. To have them share their experiences and give the long-term perspective on this calmed me down after the initial shock had settled. Then we got in touch with the other group and decided to “join forces” and approach a journal together for publication back to back.

Going this route had two main benefits:

1) the two papers would strengthen each other’s message, and
2) each team would get to sleep at night because we no longer had to worry that the other article would suddenly be out in a peer-reviewed journal and the novelty of the other study would be lost.

### Language

#### Did you encounter any challenges or any benefits of working cooperatively with people from different research groups?

Clare: I think the teams were on the same page about our goal. We were helped by mutual trust (we were not strangers) and confidence in each other’s teams (we knew both sets of data were of good quality). Our results were remarkably similar; for example, we both identified species of zoo animals as we had hoped, but both groups also identified items for the animal feed (e.g., cow, chicken, pig, and horse fed to carnivores) and animals from the surrounding wildlife (e.g., squirrels and hedgehogs). Slight differences in one relationship between variables existed; however, both teams started with different methods of collection of the eDNA and both found the exact same thing. This actually suggested that the idea was more robust than we had hoped. Not such a “crazy” idea after all.

**Figure:**
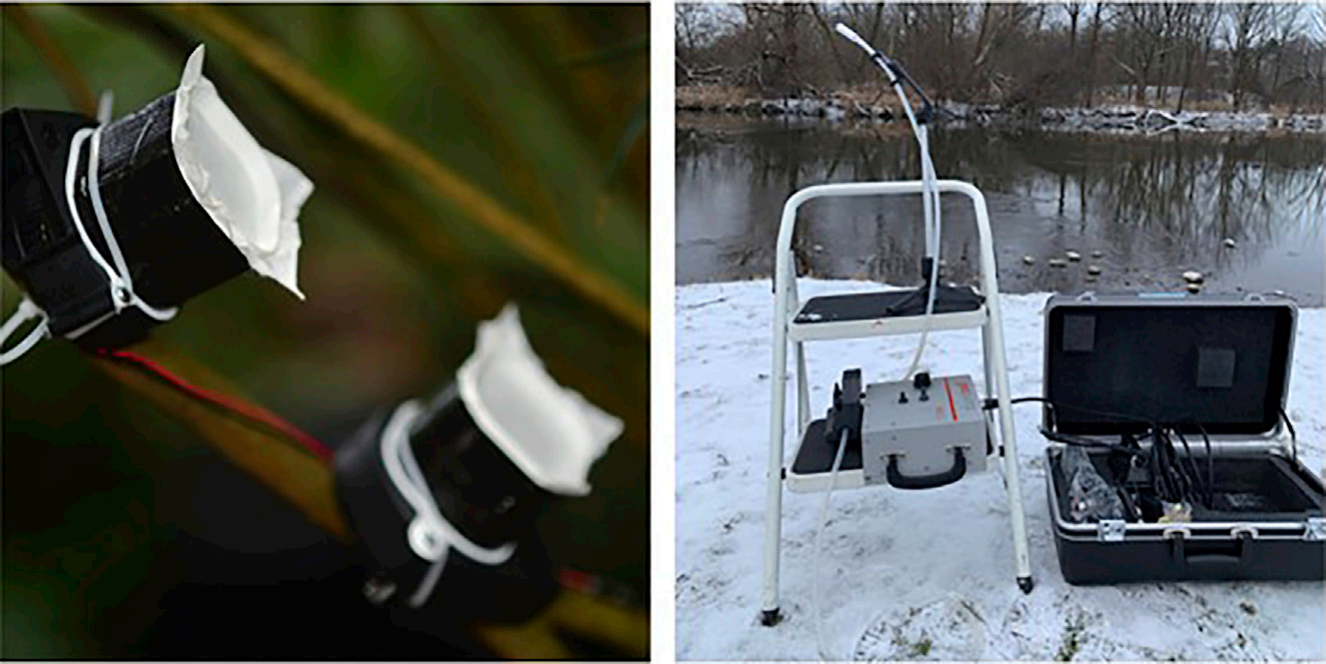
(Left) The air collection system used by the Bohmann team, Credit: Christian Bendix; (Right) The air collection system used by the Clare team, Credit: Elizabeth Clare

Bohmann: I think the ‘gentle(wo)mans’ agreement we made between the two teams when we became aware of each other’s studies meant a lot to both teams as we ventured into this very unfamiliar territory of coordinating article submission, peer-review, publication, and press with a team that we at first had seen as competitors. In addition, both teams tried their best to coordinate everything. We would even wait for each other to be ready for the different stages, e.g., submission, and be in contact so we could submit more or less at the same time.

Lynggaard: The dialogue was very good between both groups and it was easy to coordinate the submissions. I think that in this case, the impact of our studies was higher because we published both papers at the same time.

### Research methods

#### Did your cooperative approach require tailoring your research methods? How did this impact the quality of your research? How did it impact what you ultimately decided to publish?

Littlefair: The two teams worked separately until our preprints were released; so although our methods and results are similar, they were developed separately from one another.

Clare: From our side, after the initial discovery of the twin papers, we deliberately did not compare them. We did not read each other’s revisions or reviewer comments at any stage. We didn’t want them to become more similar during review, and we did not want to combine them. At one stage, an editor asked if we had considered making them into one paper. We did not want to do that. We saw real value in the replication aspect of this. I think of this as coordination, not collaboration. All scientific aspects have been totally independent.

**Figure:**
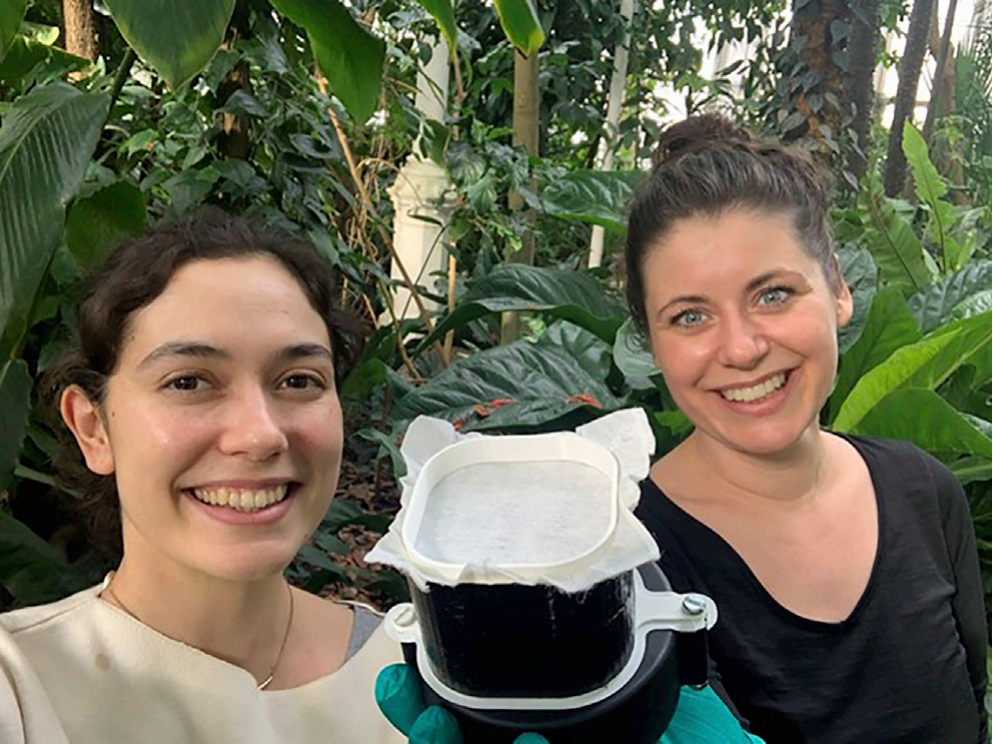
(Left) Christina Lynggaard and (right) Kristine Bohmann holding an air sampler used for sample collection

What we did coordinate was writing to editors on behalf of both teams to explain the very unusual situation. We coordinated timing, submitting the initial papers, and then revisions on the same day. One team would wait for the other to be ready so that everything was synchronized. We didn’t want anyone to “win” in the publication game. It was always about sharing the outcome. Even to the point of writing the press release to be a conversation between teams, trying our best to share any media inquiries, making sure to copy the other team on important emails, and suggesting reporters contact the other team. We think the “two papers” part of this story is as much fun as the research itself.

Bohmann: We only knew about the UK study when it turned up as a preprint. And even after knowing about the other team’s article, we did not coordinate anything regarding the actual research - for that part, each of our two teams just focused on their own studies. The only way we influenced the other team was that we agreed to cite each other’s article where appropriate. In that sense, I do not see any ethical implications - it’s also important to say that each article went through independent peer-review process.

### Governance

#### What were your concerns when you first realized your work was so similar to each other’s? What led you to take the approach that you did?

Clare: Initially … well panic! When we first realized there were twin papers we had no idea what stage the other team had reached. I have minimal experience with preprint servers and the number one worry was that if theirs had already been accepted somewhere, then ours would probably never get published. Journals always want you to explain what is novel … in this case they were so similar it would have been very hard to reframe it as anything other than a replication. It’s a funny problem. In science we are supposed to make all our procedures replicable. It’s supposed to be that way … and yet replicating someone’s work is of almost no value in the publication world where novelty wins out.

Bohmann: I do not think I have ever heard about a case where two teams joined forces when faced with competition. This was only possible because the UK team put their research out in preprint; had they not done that, we would not have known about each other’s work and we would not have been able to coordinate submission to a journal.

The reason we decided to join forces with the UK group was that we could take two routes: either join forces or race to see who would be published first in a peer-reviewed journal. If we had taken the latter approach, then even if we had gotten across the finish line first, I am not sure we would have enjoyed it as much knowing that we had then taken the novelty from the other team. In addition, there would of course be the risk that the other team would get published first. As the senior author on the Danish study, I felt a great deal of responsibility toward the postdoc, the first author of our study, and to ensure that she got as much out of her hard work on the article as possible. In that sense, it also did not feel right to gamble and try to race. Also, we have gained an enormous learning experience in collaboration rather than competition.

Lynggaard: My first concern was that one study would get more attention than the other; however, as far as I can see, both studies have gotten the same attention from both the media and the scientific community. We decided to submit together and publish together (if accepted), because it was a better use of our energy than competing against each other, which would add more stress to our lives.

Clare: Our approach - to coordinate publication was genuinely for two reasons

1) We think that for a novel proof of concept idea, independent replication is essential. No one was more surprised about how well our data turned out than us! So seeing that someone else was able to do the same thing and get the same outcome gave us confidence in what we had produced.
2) These research teams have worked together before and we hope to do so in the future. It was important to preserve that relationship, and we did not want anyone who had put so much effort into this to be left out. We wanted both teams to get recognition for what they had accomplished.

Scooping someone on an idea is not fun. In a race, someone has to lose. We did not want anyone involved to be in that situation. Working together was better for the research and better for all the people involved.

### Publication

#### How did you come to the decision to publish independently in the same journal? How has this decision impacted the reception of your work? What were the challenges during publication of this or any such research? What initiative or policy change (by publishers, funders, etc.) would make cooperative research easier/more effective?

Bohmann: Publishing in the same journal and with the same editor would enable us to control the timing of publication and press work much better. I think *Current Biology* handled the back-to-back publication so professionally that I would think it was something they encountered more often than I think is the case.

Lynggaard: Having more of these parallel studies being published could help to make cooperative research easier. Seems that not many researchers think it is a possible option and are left with the idea that the only option is competing against each other. Doing science is difficult and takes time, so it is a shame having to rush a study or leave the manuscript in a drawer when another group comes out with similar results as yours in a preprint. Cooperation and good communication can be the way forward.

Clare: It wasn’t a decision so much as the only logical thing to do. Once the decision had been made not to compete, the only alternative was to coordinate. We contacted two journals using the unorthodox approach of writing directly to the editors, one letter from both teams trying to explain how unusual it was and that our singular goal was to have two papers published together at the same time. We wrote to ask how we could submit our papers for full peer review but have them connected all the way through the process. One journal never responded. One editor agreed to read the papers but declined to review them.

It was actually someone not on either author team that wrote to *Current Biology* to explain the situation describing us as being very sensible in our approach to our problem. We weren’t even part of that initial contact but the editor gave us specific instructions on submission; for example, explicitly talking about both papers in the cover letter. After that it was just like any other peer-review experience other than that we tried to keep everything timed together.

The net impact has been phenomenal. I think the papers have gotten significantly more attention because there is a story of research and a very human story of the altruistic decisions we collectively made. Media outlets seem interested in both parts. Students, audiences, and colleagues keep asking … How did you manage to pull this off together?

I don’t think there is a policy or funding change that can encourage this. We did not (and would not) set out to do this. In most cases it would not work, and in most papers there would not be much value in this degree of replication. It did slow down the process of publication so there was a cost. I would not want to see journals start to encourage copies of research. In general, I think we are better to put our efforts into expanding knowledge, not replicating it. But in some cases, such as validating a totally new idea that has strong potential to transform a discipline, I think the replication is extremely valuable.

Littlefair: There are some journals which specifically value replicated results or will consider your paper even if there is not a high degree of novelty. Typically these studies come out after the publication of the first paper and not simultaneously like us! But although there is pressure to publish high impact papers and publishers value novelty, it is difficult to invest time into replicated studies, even when these are so valuable for our science. In the field of aquatic eDNA, studies on the same phenomena (e.g., linking eDNA release to the abundance of organisms) are really important because this builds up our datasets to allow us to do meta-analyses. There are so many environmental variables affecting eDNA phenomena that replication and eventual meta-analysis will be crucial for teasing apart the really important factors which shape eDNA distribution in natural habitats.

I think, in addition to formal policy initiatives, informal culture change is necessary as we learn to love replication. Instead of talking to your lab about the secrecy of your results and how afraid you are of being scooped, we can talk about the power of large collaborative teams and the necessity of replication to move science forward.

### Conclusion/final thoughts

#### What did you learn about taking a cooperative approach to navigate overlapping work and what tips would you give to anyone who finds themselves in the same situation? Are there any other challenges you encountered that aren’t discussed here?

Bohmann: It will be a case-by-case judgment of which route would be best to choose: racing or joining forces. When joining forces, I think the most important thing is trust, having a common goal and that both teams see the purpose of going down this route; and then, of course a good editor and journal.

Lynggaard: I agree with Kristine; not all cases are the same. If trust is not there, cooperation will be difficult.

Littlefair: I was surprised and really pleased by how positive this experience has been. I think the suggestion from Beth to reach out to the Danish team was brave but paid off very well. By reaching out to talk to Kristine and Christina we found common ground rather than thinking of them as our rivals, which is so often the way that young scientists are taught to navigate these situations. I remember people not talking about their results at conferences or keeping parts of their methods hidden so they wouldn’t be scooped. I didn’t ever receive any input about how positive working with your “rivals” could be.

Clare: I agree with Joanne. When we learned of the “other” paper, I contacted colleagues for advice. There was only one answer that made any sense: Call Denmark. So we called. We have been extremely pleased with how this came together. We have been far more successful as two teams coordinating than two teams competing. I have lectured my undergraduate classes about this case. I have told the story of the two teams to reporters and colleagues. The general response has been “but that’s how science should work!”
